# Cut it and forget it: Can patient agency go too far?

**DOI:** 10.1016/j.hrcr.2023.08.010

**Published:** 2023-08-24

**Authors:** Alexander A.W. Leunig, Shalini D. Allam, Alaa A. Shalaby, N.A. Mark Estes

**Affiliations:** University of Pittsburgh Medical Center, University of Pittsburgh School of Medicine, Pittsburgh, Pennsylvania

**Keywords:** Chronic pocket infection, Pocket erosion, Twiddler’s syndrome, CIED, ICD

**Key Teaching Points**

• Implantable cardioverter-defibrillator implant pocket erosions can occur years after initial implant.
• Patients can present with a high level of denial of their situation regarding pocket erosions.
• Nonsuicidal extreme manipulation of the leads is possible, even up to the point of cutting of the leads and “twiddling” with the leads.
• Regular follow-up and patient education are needed even years after implant to recognize and prevent pocket erosions and treat possible twiddler’s syndrome.

## Introduction

We present a case and images of a patient who used kitchen shears to cut off both leads of his 8-year-old implantable cardioverter-defibrillator (ICD) implant after it eroded through his skin. This case presents an unusual combination of pocket erosion, overwhelming denial, and patient manipulation of the leads. It also illustrates the complex psychology associated with ICD implant, the perpetual risk of associated infection, and the continued need for patient education and follow-up long after initial implant.

## Case report

A 66-year-old male patient with a history of coronary artery disease, heart failure with reduced ejection fraction, and atrial fibrillation was transferred to our facility with concerns for sepsis, secondary to device infection. Eight years prior to presentation he had received a left-sided dual-chamber ICD for primary prevention of sudden cardiac death. He was unaware of any therapy by the device. Since the beginning of the pandemic, he had been lost to follow-up.

Several months prior to this presentation, our patient noted a small skin defect at the left lateral chest border of his ICD pocket. Over the following weeks and months the patient’s attempts to treat the defect with topical antibiotic creams and bandages were unsuccessful. The defect became progressively larger, draining purulent and foul-smelling bloody yellow fluid. By his report, approximately 2 months before admission to our facility, the defect became so large that the pulse generator spontaneously fell out of the pocket during a bandage change. At this point—with the generator only hanging on by its 2 leads—the patient cut both leads off with kitchen shears roughly 4 cm from the pulse generator itself. He then disposed of the generator and the attached leads in the kitchen garbage. He buried the sheared lead ends in the pocket and continued to apply bandages and cream over the defect.

Starting 2 weeks preceding this admission he developed fever, chills, and increased confusion. His wife, who was not aware of any of the events above, called emergency medical services, seeing his worsening symptoms and lethargy.

**Figure 1:**
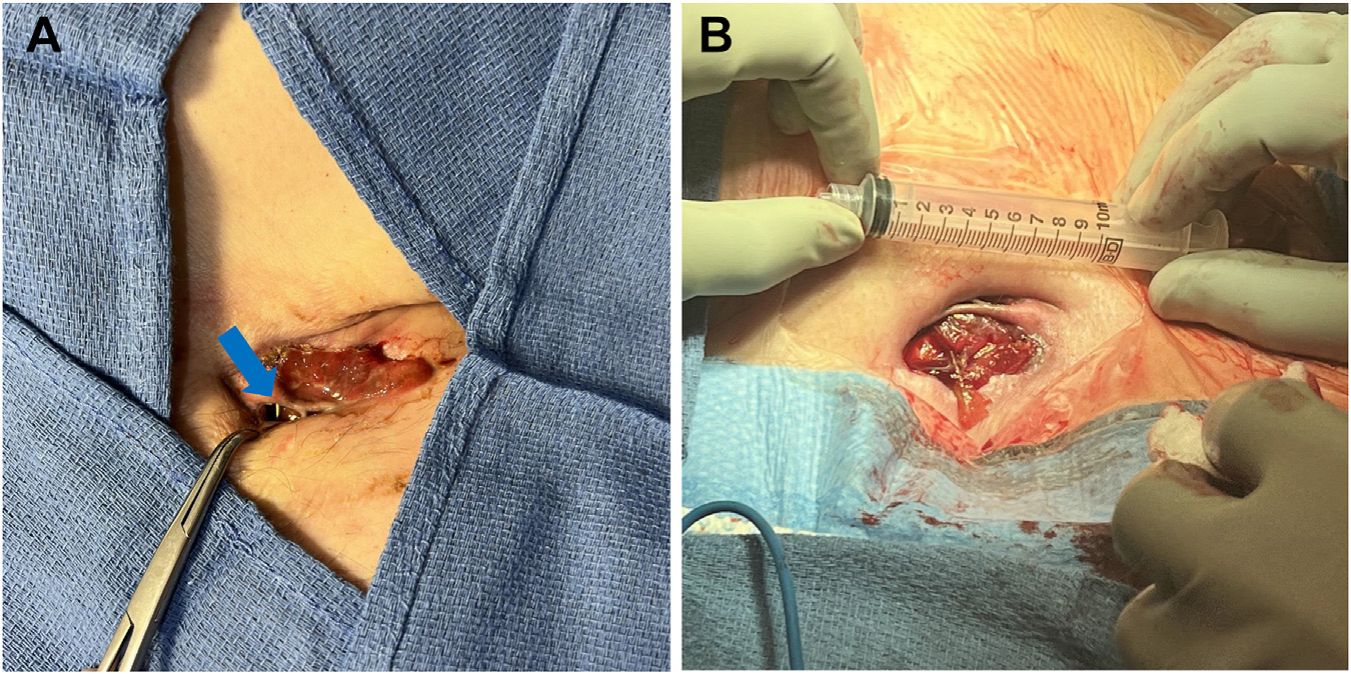
**A:** Photograph of the chest wound with leads exposed at bedside (*blue arrow*). **B:** Intraoperative picture of the wound with scale after extraction.

Upon admission, our patient presented with a 7 × 2 cm wound on his left upper chest, with yellow-brownish drainage, but little edema or erythema. The severed leads were visible upon slight retraction of the wound edges (Figure 1). Notably, the leads were eroded out of the skin caudally and lateral to the insertion site. The patient denied suicidal ideation at any point during his disease course. The patient’s mood and judgment were assessed to be intact. When asked, he stated he did not want to bother his wife with his health issues and was reluctant to make medical appointments since the early days of the pandemic.

**Figure 2:**
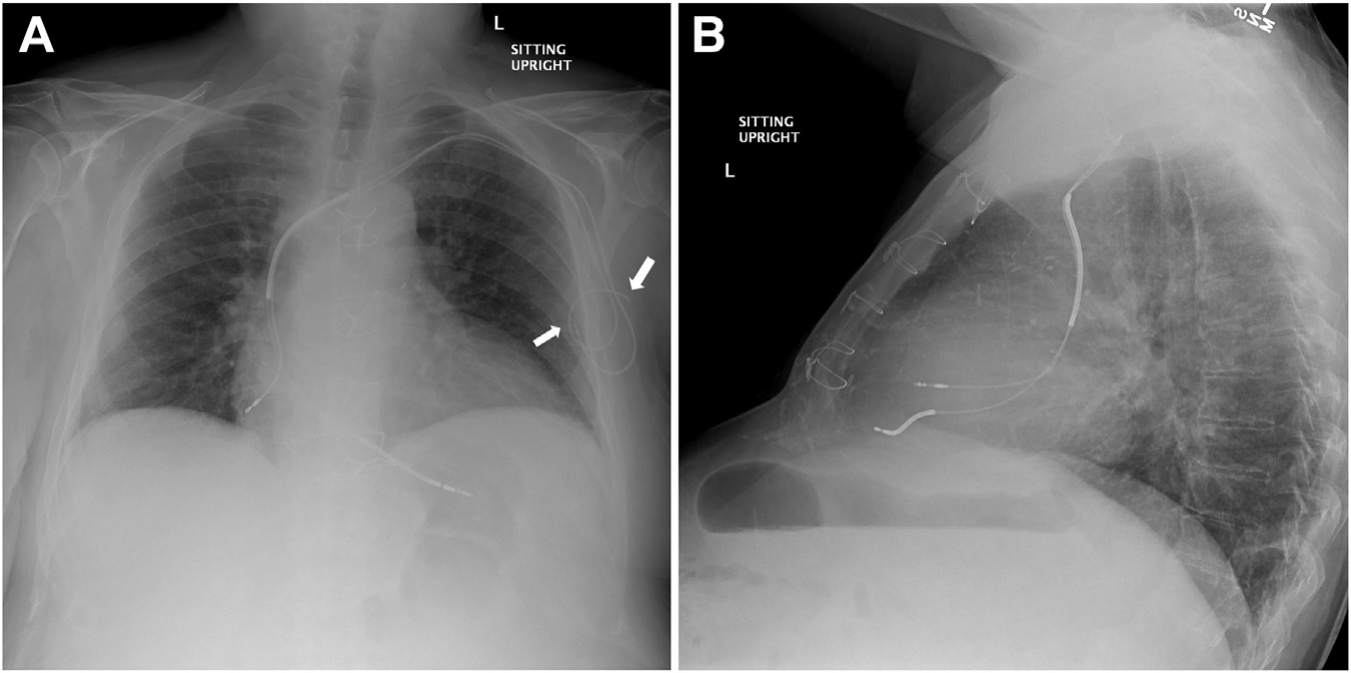
**A:** Posteroanterior chest radiograph. The severed leads are marked by white arrows. **B:** Lateral chest radiograph.

On chest radiograph the severed leads appeared to be slightly retracted, with good-sized lead remnants in the pocket (Figure 2). The leads still terminated in the right atrial appendage and the right ventricle. Notably, there was little slack on either lead. Prompt antibiotic treatment with ampicillin/sulbactam, initiated after blood cultures yielded *Proteus mirabilis*, improved the patient’s clinical status. After discussion during interdisciplinary endocarditis rounds, the patient was scheduled for lead extraction. After successful extraction of the leads, the wound was debrided and left open to heal by secondary intention. The patient was subsequently discharged in stable condition.

## Discussion

Our case is unusual in more than 1 aspect. First, other than a report of attempted suicide by cutting pacemaker leads,^1^ our case is a unique report of nonsuicidal removal of the pulse generator and cutting of the leads.

Secondly, this patient-initiated event was preceded by a pocket infection that led to the complete exposure of the device. Pocket erosions are likely owing to indolent infection usually acquired at the time of implantation.^2^ In this case well over 7 years had elapsed since implant. Other possibilities include hematogenous seeding during episodes of transient bacteremia or trauma. Our patient could not offer supportive history for either possibility. The fact that the erosion grew to the point the device would fall out before the patient sought medical attention and his decision to take matters into his own hands speaks of a high level of denial. Given the overwhelming denial, this pocket infection could indeed have been smoldering for years.

Thirdly, there was little slack on either lead, as seen on the chest radiograph. This could be owing to 3 reasons: (1) there was no slack at the time of implantation, (2) the generator falling out created excess traction, or (3) the patient twiddled with the exposed leads or the pacemaker. Usually, patients with twiddler’s syndrome deny or are oblivious to their manipulation. While the tell-tale lead entanglement noted in such cases was absent on current radiography, prior radiographs were not assessed and therefore cannot be excluded.

Indeed, in extreme cases twiddler’s syndrome can lead to erosion of the pacemaker pocket and lead malfunction.^3^, ^4^, ^5^ Although the patient did not directly manipulate the pacemaker before externalization, he did manipulate the wound, the pacemaker, and the leads after they fell out. Unmistakably, denial was operative in our patient’s response to the erosion, its progression, and his decision to remove the device without letting anyone know. Could this indeed have been an extreme case of twiddler’s syndrome leading to or spurred by device pocket infection? Our patient was fortunate not to be pacemaker dependent, and the lead extraction was performed safely and successfully.

In any case, we believe this report illustrates the complex psychology associated with ICD implants, the need to educate patients about the importance of regular follow-up, and the ever-present risk of device infection long after initial implant.
